# Dementia-Associated Compulsive Singing (DACS): Presentation of Unpublished Clinical Cases Miniseries

**DOI:** 10.3390/ijerph191710844

**Published:** 2022-08-31

**Authors:** Roberto De Masi, Stefania Orlando, Maria Carmela Costa

**Affiliations:** 1 Complex Operative Unit of Neurology, “F. Ferrari” Hospital, Casarano, 73042 Lecce, Italy; 2 Laboratory of Neuroproteomics, Multiple Sclerosis Centre, “F. Ferrari” Hospital, Casarano, 73042 Lecce, Italy; 3 Complex Operative Unit of Ophthalmology, “V. Fazzi” Hospital, 73100 Lecce, Italy

**Keywords:** L-dopa, punding, compulsive singing, dementia, dopaminergic circuit

## Abstract

Dementia-associated compulsive singing (DACS) is a neurotransmettitorial-based behavioral disturbance, characterized by an unabating melodic expression, occurring in patients that suffer from evolved dementia. Previously described only as a “punding” aspect of the dopamine dysregulation syndrome (DDS) in the Parkinson’s disease (PD), compulsive singing has now been described, for the first time, in four non-PD patients effectively treated with Haloperidol or Quetiapine. Unlike the DDS-associated conditions, in our cases DACS is not pharmacologically induced, being that all patients were L-dopa-free. We detected a diffuse hyperintensity of the white matter and brain atrophy, with insular shrinkage as well as ventricular system and/or sub-arachnoid space enlargement in our DACS patients. Furthermore, similarly to the other behavioral symptoms of dementia, DACS also seems to be correlated to the degree of cognitive and functional impairment, rather than its subtype. In conclusion, DACS is a non-cognitive, unpublished clinical aspect of evolved dementia, which is interesting due to the involvement of the extra-nigral dopaminergic system, resulting in an unabating altered behavior, but also to the enrichment of our knowledge in the involutional diseases of the central nervous system and their physiopathological manifestations.

## 1. Introduction

Dementia-associated compulsive singing (DACS) is a neurotransmettitorial-based behavioral disturbance, characterized by an unabating melodic expression, out of context and devoid of information content, occurring in patients suffering from evolved dementia. Previously described only as a “punding” aspect of the dopamine dysregulation syndrome (DDS) [1,2,3,4,5,6] in Parkinson’s disease (PD) [7,8,9,10], compulsive singing (CS) has now been described in four non-PD patients for the first time. 

DACS core features refer to an infrequent condition occurring at a late age without discrimination regarding gender, characterized by a good response to Haloperidol and Quetiapine, excluding Benzodiazepine and Trazodone, proving this condition to be dopaminergic in nature. The frequently associated conditions are both a low level of education and employment, and a low socio-economic extraction, in the absence of a clear premorbid personality or a suggestive lifestyle.

Traits of DACS are of the sub-cortical or cortical type of the impaired cognition with prevalent ideomotor slowdown and impoverished speech, respectively. Depending on the pre-existing grade of cognitive impairment, we noted a vocal expression ranging from a structured word melody to a chant or unstructured lament. In all case, concomitant reversible failures in the environmental contact increase, resulting in a worsening of relation functions. Moreover, the DACS onset and its evolution seem to be affected by intercurrent medical conditions, such as fever and infections. Diffuse hyperintensity of the white matter and brain atrophy, with ventricular system and/or sub-arachnoid space enlargement were also detected, as well as a low score in the Barthel index [11] and the common non-applicability of neuropsychological tests. Vascular comorbidities of DACS are more frequent with associated vascular type dementia, rather than the degenerative type, as expected. Finally, unlike DDS acting pharmacologically on the nigro-striatal system of PD-patients [12], DACS expresses endogenic extra-nigro-striatal involvement, being L-Dopa-free in all patients considered until now. 

In conclusion, DACS is an unpublished novel clinical finding, which is interesting to enrich our knowledge about involutional diseases of the central nervous system (CNS) and their physiopathological manifestations. 

## 2. Cases Presentation

### 2.1. Patient 1

Patient 1 is D.G.P., an 84-year-old farmer and quiet inhabitant of the southern outskirts of the Salentinian Peninsula in southern Italy. After the third year of study, he dropped out of elementary school to be employed in the fields and has always led a quiet life thereafter. In addition, he has never manifested any behavioral, thought or mood disturbances. 

About ten years ago, he fell ill with hypertension, treated with 10 mg daily of Ramipril and statin-treated hypercholesterolemia. However, already one year before, the patient had experienced the onset of progressive short-term memory disturbances. Over the years, especially the last two, he has also manifested concrete thinking and reduced verbal and motor initiative, poor language and dependence for activities of daily living (ADL). Only a few comorbidities were reported, such as the moderate hypercholesterolemia and chronic ischemic heart disease, without rhythm abnormalities. 

In 2011, he received the diagnosis of Alzheimer disease (AD) at the AD Evaluation Unit of the “F. Ferrari” Casarano Hospital, with a prescription of 8 mg Rivastigmine transdermal treatment. The neurological examination evidenced no focal deficits, but a global reduction in the motor performances with a precautionary gait. At the moment, the segmentary force results proportional to the muscle trophism with a conserved tone. Tendon reflexes are present and symmetric. Intrinsic and extrinsic ocular motility as well as cranial nerves are in order, too. No evidence of trochlea dentata or other movement disturbances were noted, with the only exception of a mild stance in anterior flexion of the trunk.

Apart from memory loss, the psychic examination revealed a concentration deficit and hypoposexia, with ideomotor slowdown, mild bradykinesia, and temporal-spatial disorientation. As part of a reduced motor initiative, the patient currently spends his life predominantly sitting and, in the last month, also singing all the time, without talking. Specifically, the singing consists in the reproduction of one or more known melodies sometimes accompanied by the words, which may be the original ones or totally invented ones. More frequently, the singing resembles a persistent chant expressed in a stereotypical and repetitive way, without any mood resonance, grimace or non-verbal communication. Furthermore, it is also present during some activities, such as micturition, deambulation, etc. As regards his mental state, we noted a reduced surrounding environmental contact and a global inattention during the singing, with a transient and short restoration in relation functions, through the intense verbal or touch stimulation of the patient. 

**Figure 1:**
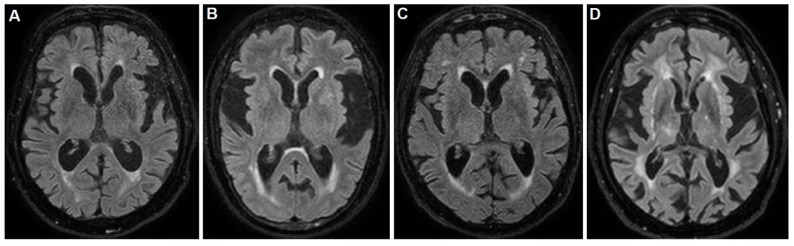
Brain magnetic resonance imaging (MRI) short tau inversion recovery (STIR) sequences of (**A**) patient 1: note the enlargement of the ventricular system and subarachnoid spaces with mild hyperintensity of the occipital horns periventricular white matter; (**B**) patient 2: note the enlargement of subarachnoid spaces with diffuse deepening of the cortical sulci and a thinning of the cortical mantle, especially at the fronto-temporal lobes. The hyperintensity of the occipital horns periventricular white matter is also visible; (**C**) patient 3: note the slight diffuse shrinkage of the subcortical grey matter with several hyperintense lesions at semioval centers; (**D**) patient 4: note the subcortical chronic ischemic lesions with vascular encephalopathy and periventricular diffuse hyperintensity. Note the atrophy of insula as a common feature of these brains.

The neuropsychological tests, including the Mini Mental State Examination (MMSE) [13], were not applicable and the Barthel index was 25/100. Brain magnetic resonance imaging (MRI) evidenced the insular atrophy, with greater left expression, the enlargement of the ventricular system and subarachnoid spaces with mild hyperintensity of the periventricular white matter, as shown in Figure 1A. 

After the neurological consult, the patient underwent a Haloperidol 2% solution, at a 0.5 mg dosage three times daily, with a dramatic improvement, while no benefit was found with Trazodone and Benzodiazepine.

### 2.2. Patient 2

Patient 2 is M.G., a 78-year-old obese woman who has always been a housewife and devoted Christian believer. She completed elementary school and subsequently devoted herself completely to housework. She has never experienced thought or mood disorders either at a young age or in adulthood. 

The subject had good health until the age of 70, when she developed a progressive cognitive deterioration. In particular, the first sign was difficulties in handling objects and clothing; in fact, she had to be helped when dressing and using common objects. About one year later, the patient also manifested a slight speech disorder with trouble in naming objects and faces, as well as the use of circumlocutions to express simple concepts. Over time, reading and calculation disorders were also associated, with impossibility to manage bills, shopping and even trivial purchases. Finally, structured polysensory hopelessness arose too, associated with high emotional-affective resonance. 

Although these disturbances were not serious at the onset, they had a progressive course, until the patient was completely dependent in all ADL. Reported comorbidities are hypercholesterolemia, chronic ischemic heart disease, arterial hypertension treated with 5 mg daily of Amlodipine and type II° diabetes in mixed therapy with 1000 mg Metformin twice daily and subcutaneous Glargine Insulin. The patient was also taking daily 100 mg of ASA and 20 mg Atorvastatin. No ictal event was reported or documented. 

In 2014, she received the diagnosis of AD at the AD Evaluation Unit of the “F. Ferrari” Casarano Hospital, with a prescription of 8 mg Exelon transdermal treatment. The neurological examination evidenced constructive and clothing apraxia as well as transitive gesture apraxia. The language evidenced phonemic and semantic paraphasias associated with prosopagnosia. The calculation capacity was reduced until to overt dyscalculia and dyslexia. The mood was quite preserved with a slight secondary flexion and no movement disturbances or their signs were evident. The psychic examination also revealed a temporal-spatial disorientation with short-term memory loss, a concentration deficit and hypoposexia, without ideomotor slowdown nor bradykinesia. The patient spent her life mainly seated watching television until about six months ago, when she gradually began to sing. Specifically, the singing consisted in the continuous reproduction of invented melodies accompanied by words on variable religious themes (Audio S1 in Supplementary Materials). The singing resembles a persistent chant expressed in a poor language in repetitive way and, partially, in local dialect, with a mood resonance and accompanying facial grimaces also interfering with some activities, such as eating and speaking. In fact, these activities become temporarily possible only after drastic shaking or algogenic stimulation of the patient leading to a concomitant short restoration in relation functions, as the mental state was characterized by a reduced environmental contact and a global inattention during the singing. 

The MMSE, as well as other neuropsychological tests, were not applicable and the Barthel index was 25/100. Brain MRI evidenced the insular atrophy, with greater left expression, the enlargement of subarachnoid spaces with diffuse deepening of the cortical sulci and a thinning of the cortical mantle, especially at the fronto-temporal lobes, as shown in Figure 1B. After several weeks from the singing onset, the patient underwent a Trazodone and Benzodiazepine based therapy, without any improvement. Finally, she underwent the Haloperidol 2% solution, at a dosage of 0.5 mg three times daily, with a dramatic improvement. 

The consultant neurologist was R.D.M., the same one treating the case 1. Unfortunately, the follow-up was very short, because the patient died a few months after the drug initiation, due to sudden death. No specific therapy was started for dementia. 

### 2.3. Patient 3

Patient 3 is F.E., an 83-year-old man, merchant and agricultural entrepreneur of lower Salento in southern Italy. Despite his low educational level (incomplete primary school), he entertained working and commercial relationships with many local people, without ever manifesting mental disorders or behavioral abnormalities.

He was in good health until five years ago, when he developed a progressive cognitive deterioration. At that time, he was already affected by arterial hypertension, treated effectively with 20 mg daily of Olmesartan. The first pathological manifestation was anxiety and structured polysensory hopelessness, associated with high emotional-affective resonance and agitation. In addition, increased behavioral disturbances secondary to an abnormal ideation with suspiciousness and fear of being poisoned or beaten. 

However, cognitive disturbances were not severe as indicated by the MMSE score of 23.4 (corrected for age and education). Two year later, the patient also manifested a weakness of the lower limbs, due to the spondylarthrosis and related myelopathy from stenosis of the medullary canal, resulting in wheelchair usage and sphincteric disturbances with recurrent urinary tract infections. Although these cognitive disturbances were not serious at the onset, having a slow progression, the patient reached an overall inability and became completely dependent in all ADL. Recently he has received the diagnosis of vascular dementia (VD) at the AD Evaluation Unit of the “F. Ferrari” Casarano Hospital, with a prescription of two daily doses of 5 mg Donepezil. 

The neurological examination evidenced temporal disorientation, concrete thinking and language disturbances (anomies, naming errors, partial understanding of complex orders, poor language) as well as a paresis on the lower limbs; in addition, behavioral disturbances associated with unstructured delirium of poisoning and noxiousness. These conditions have recently worsened in conjunction with urinary tract infection and fever, resulting in increased agitation, confusion and persistent singing. The latter reproduces well-known motifs from his youth as persistent chants expressed in a repetitive way, with a mood resonance and facial grimaces, which also interfere with some daily activities, such as eating, current speaking and interacting with the surrounding environment. The mood was quite preserved and no movement disturbances or their signs were evident. 

During this worsening phase, the MMSE, as well as other neuropsychological tests, were not applicable. The Barthel index score was 20/100. Brain MRI evidenced a slight diffuse shrinkage of the subcortical grey matter with several hyperintense lesions at semioval centers and the insular atrophy, with greater left expression, as shown in Figure 1C. Ten days after the singing onset, the patient underwent hospitalization due to the dyspnea and respiratory insufficiency. In this case, the patient already was taking 10 mg daily of Memantine, 75 mg daily of Quetiapina in refracted dose manner and 1 mg three times daily of Haloperidol, due to his deliriant state. However, the therapeutic response of the singing was poor, and the patient developed a respiratory insufficiency with hospitalization and loss to follow-up. The consultant neurologist was R.D.M.

### 2.4. Patient 4

Patient 4 is M.B., an 81-year-old housewife of southern Italy, who recently arrived in the emergency room of the “F Ferrari” hospital, already affected by evolved dementia. She was taken to the local emergency service for a rapid worsening of the cognitive impairment and loss of independence.

The patient was singing during the whole stay, and some anamnestic data was collected from a niece. Due to archaic traditions, she was withdrawn early from the elementary schools and routed to a simple life, dedicated first to her familiars and after to her husband. The remote pathological history revealed an isolated period of juvenile depression, but no subsequent mood or formal thought disorders have ever been reported. 

Moreover, a well-controlled arterial hypertension emerged, treated with 5 mg daily of Amlodipine; chronic obstructive pulmonary disease (COPD) without recurrence; polyarthrosis and, indeed, dementia for 5–6 years. Routine blood tests evidenced a mild neutrophilic leukocytosis (12,000 WC, 80% neutrophil count) and newly founded kidney insufficiency, with 65 mg% hyperazotemia. The patient had already been singing for about ten days with a continuous chant of incomprehensible words, and an invented melody often resembling a lament. 

The MMSE, as well as other neuropsychological tests, were not applicable. The Barthel index score were 10/100. Brain MRI evidenced a subcortical chronic ischemia and vascular encephalopathy with periventricular diffuse hyperintensity, as well as the atrophy of insula, as shown in Figure 1D. The neurological examination found difficult access to the patient, due to the singing and a severe deterioration in all superior cortical functions, resulting in aphasia, agnosia and apraxia, configuring the so-called alogic syndrome. In addition, slight spastic rigidity in all limbs and poor environmental contact were noted. After infusional rehydration, the 5 mg daily Haloperidol therapy greatly reduced the singing. However, the other cognitive disturbances persisted with apathy and poverty of speech and its contents. Finally, the patient was sent to a territorial rehabilitation treatment and assistance path with a prescription of 10 mg daily of Memantine. 

The legal guardian of this patient as well as those of other patients here reported, subscribed the informed consent for the description and publication of the case. 

## 3. Conclusions

It is known that non-cognitive, behavioral disturbances constitute a large component of the dementia syndrome. They correlate with the degree of cognitive impairment, irrespective of its subtype [1]. In this light, we described here four unpublished cases of CS, all associated with non-PD evolved dementia and effectively treated with Haloperidol or Quetiapine. 

The study of this phenomenon, singular as it is, represents not only a scientific curiosity, but it also increases our knowledge regarding behavioral phenomena and their underlying mechanisms, which is the aim of the present work. 

Precisely for this purpose, considering the behavioral nature of the disturbance that makes it unreproducible and non-standardized, one might think that the low sample size is an affecting bias. However, given the rarity of the form and its clinical coherence in the described cases, this concern is less relevant, but it constitutes a study limitation regardless.

Previously described only as a “punding” aspect of the PD, CS is thought to be induced by the excessive functional enhancement of the nigro-striatal dopaminergic inhibitor system, deriving from an exceeding dosage in dopamine replacement therapy (DRT), an additive pattern, in turn, of the DDS [2]. This pathological postulate of CS is shared with other compulsive vocalization disturbances, including the vocal tics of Tourrette’s syndrome and klazomania [14,15]. Consistently, these conditions recognize a common antidopaminergic intervention, based on the neuroleptic agent usage, as Haloperidol and Quetiapine. However, the lack of sensitivity of some forms suggests a pathological mechanism other than the DDS one. This is the case of DACS too. In our most likely hypothesis, DACS is due to a relative prevalence in dopaminergic activity compared to the impaired cholinergic one. Accordingly, only one patient underwent anticholinesterase therapy. Based on this neurophysiological consideration, we used the Haloperidol as a first therapeutic attempt, given its great antidopaminergic profile [16]. In fact, unlike the DDS-associated conditions, DACS although responding to the neuroleptic therapy is not pharmacologically induced, being that all the described patients were L-dopa-free.

Specifically, DACS could be characterized by an endogenic imbalance of the extra-nigral Dopamine (Da)/Acetylcholine (Ach) system, in favor of the dopamine, at the mesolimbic-mesocortical system. In fact, cognitive decline is associated with Ach impairment, but the sensitivity of DACS to the neuroleptic agents also suggests its dopaminergic nature. As is well known, the extra-nigral dopaminergic system involves motivation, cognitive processes, iterative behaviors, and emotional states through the mesolimbic pathway, as well as the executive functions, attention, working memory, inhibitory control and planning through the mesocortical one [17]. Coherently with its extra-nigral dopaminergic nature, core features of DACS reflect the mesolimbic-mesocortical involvement, expressing an unabating melody in demented L-Dopa-free patients. Depending on the pre-existing cognitive impairment, this vocal expression ranges from a structured word melody to a chant or unstructured lament with a concomitant reversible failure in the environmental contact, and finally, in a worsening of social relation functions. 

A good response to Haloperidol, excluding Benzodiazepine and Trazodone, is a common finding too. On the other hand, the observations in favor of Quetiapine are too small to be considered more than anecdotal. In any case, Quetiapine and other atypical neuroleptics are to be considered, in our opinion, as low-potency and second-line therapy of DACS, due to their receptor profile less selective for Dopamine [18,19].

Moreover, similarly to most neurological diseases, including dementia, DACS onset and evolution seem to be affected by intercurrent medical conditions, such as fever and infections as enhancing or determining factors. 

Diffuse hyperintensity of the white matter and brain atrophy, with ventricular system and/or sub-arachnoid space enlargement, mostly in the insula (with greater left expression), are detected in DACS patients, although vascular comorbidities are more frequent with associated vascular type dementia, rather than the degenerative one, as expected. Furthermore, the involvement of the insula is a notably and interesting finding in the present context, as this anatomical structure, which is part of the quadrilateral space of Pierre Marie, is in charge of functions related to emotional expressions, including empathy, environmental perception, motor control, self-awareness, cognitive functioning, interpersonal experience, and emotional homeostasis [20]. All these functions can be affected in the evolved dementia, and considered putative DACS contributors. This is consistent with the known role of the insula in mediating the motor aspects of speech production, in particular the articulatory ones but also the dopamine-dependent executive abilities [21].

Common vascular comorbidities associated with DACS are hypercholesterolemia and hypertension. Type I° or II° diabetes mellitus is another metabolic comorbidity. A low score of the Barthel index and the common non-applicability of neuropsychological tests evidence a high degree of mental and functional impairment associated with DACS. Specifically, DACS patients present an important dysexecutive syndrome with hypoposexia and reduction in environmental contact as well as in the field of consciousness. This condition appears to be sensitive to tactile or verbal stimuli administrated by the operator, resulting in the transient recovery of visual contact with short responses, the content of which depends on the preexisting degree of cognitive impairment. 

In fact, tactile or verbal stimuli can transiently ameliorate the environmental contact in DACS patients, providing a putative non-pharmacological approach. However, although this physical intervention appears currently unpractical, it should be considered in a future rehabilitative perspective. Arthrokinetic, tactile and verbal stimuli may also be responsible for the prevention of DACS, all of which are activities known to preserve synapses and plasticity [22,23,24,25,26]. Consistently, due to the low educational level, our patients have of a small cognitive and functional reserve.

Finally, similarly to the other behavioral symptoms of dementia, DACS also seems to be correlated to the degree of cognitive and functional impairment, rather than its subtype. Table 1 resumes clinical remarks of the four patients and core features of DACS.

In conclusion, DACS is a non-cognitive, unpublished clinical aspect of evolved dementia, which is interesting due to its involvement of the extra-nigral dopaminergic system and the insula, resulting in an unabating altered behavior, but also to the enrichment of our knowledge in the involutional diseases of the CNS and their physiopathological manifestations.

## Figures and Tables

**Table 1:** The clinical features of four unpublished cases affected by DACS.

	CASE 1	CASE 2	CASE 3	CASE 4
**T** **ype of Dementia**	Degenerative sub-cortical	Degenerative cortical	Vascular	Vascular
**MMSE score**	Not applicable	Not applicable	23.4	Not applicable
**Neuropsychological deficit**	Dysesecutive syndrome, ideomotor slowdown	Higher cortical functions involvement	Language and behavioral disturbances; unstructured delirium	Alogic syndrome
**Barthel index score**	25	25	20	10
**Comorbidity**	Hypercholesterolemia, chronic ischemic heart disease	Hypercholesterolemia, chronic ischemic heart disease, arterial hypertension, DMII	Arterial hypertension with labile therapeutic compensation	Arterial hypertension, COPD, polyarthrosis
**DACS therapy**	Haloperidol 0.5 mg three times daily; Non effective Trazodone and Benzodiazepine	Haloperidol 0.5 mg three times daily; Non effective Trazodone and Benzodiazepine	75 mg of Quetiapine daily and 0.5 mg of Haloperidol three times daily (poor efficacy in conjunction with fever)	5 mg daily of Haloperidol with efficacy after infusional rehydration
**Neuroimaging**	Ventricular and subarachnoid space enlargement, insular atrophy with greater left expression	Thinning of the cortical mantle, especially at the fronto-temporal lobes, insular atrophy with greater left expression	Slight diffuse shrinkage of the subcortical grey matter, hyperintense lesions at the semioval centers, insular atrophy with greater left expression	Chronic subcortical ischemia with periventricular diffuse and some focal hyperintensity, insular atrophy
**DACS features**	Persistent chant without mood resonance	Persistent chant, poor language with mood resonance	Persistent chant, poor language with mood resonance	Persistent chant with incomprehensible words resembling a lament
**E** **ducational level**	Incomplete primary school	Primary school	Incomplete primary school	Incomplete primary school
**O** **ther drugs**	Atorvastatine 20 mg daily; ASA 100 mg daily	Amlodipine 5 mg daily; Metformine 1000 mg twice daily; Glargine Insulin s.c. 18 IU daily; ASA 100 mg daily; Atorvastatine 20 mg daily	Olmesartan 20 mg daily	Amlodipine 5 mg daily

**MMSE** mini mental state examination; **DACS** dementia-associated compulsive singing; **COPD** chronic obstructive pulmonary disease; **DMII** type two diabetes mellitus; **ASA** acetylsalicylic acid; **s.c.** subcutaneous injection; **IU** international unit.

## Data Availability

Not applicable.

## Supplementary Materials

The following supporting information can be downloaded at: https://www.mdpi.com/article/10.3390/ijerph191710844/s1, Audio S1: representative recording of a patient’s compulsive singing described in our work.
